# CD9 expression as a favorable prognostic marker for patients with malignant mesothelioma

**DOI:** 10.3892/or.2012.2116

**Published:** 2012-10-31

**Authors:** VISHWA JEET AMATYA, YUKIO TAKESHIMA, KEISUKE AOE, NOBUKAZU FUJIMOTO, TOSHIHIRO OKAMOTO, TAKETO YAMADA, TAKUMI KISHIMOTO, CHIKAO MORIMOTO, KOUKI INAI

**Affiliations:** 1 Department of Pathology, Institute of Biomedical and Health Sciences, Hiroshima University, Hiroshima; 2 Department of Medical Oncology, Yamaguchi-Ube Medical Center, Yamaguchi; 3 Department of Respiratory Medicine, Okayama Rosai Hospital, Okayama; 4 Division of Clinical Immunology, Advanced Clinical Research Center, Institute of Medical Science, University of Tokyo, Tokyo; 5 Department of Pathology, Keio University, Tokyo, Japan

**Keywords:** CD9-shRNA, migration, CD9 immunohistochemistry, mesothelioma, survival

## Abstract

CD9 is involved in cell growth, adhesion and motility and its expression is reported to be of prognostic significance in various types of human malignancies. We found increased cell migration in the mesothelioma cell lines MSTO-211H and TUM1 following *in vitro* shRNA-mediated knockdown of CD9 expression. We investigated CD9 expression in 112 malignant pleural mesotheliomas. CD9 expression was observed in 62 of 71 epithelioid, 13 of 20 biphasic and only 1 of 21 sarcomatoid mesotheliomas. Among the epithelioid mesotheliomas (EMs), CD9 expression was observed in all of the 33 cases with a differentiated type (EM-D) and in 29 of the 38 cases with a less-differentiated type (EM-LD). Patients with CD9 expression showed higher 1- and 2-year survival rates (63 and 25%) compared to the patients without CD9 expression (39 and 11%). Univariate analysis revealed that patients with CD9 expression demonstrated a more favorable survival (P=0.0025) along with other clinicopathological factors, including age younger than 60 years, IMIG stage I–II, epithelioid histology, EM-D and patients who underwent extrapleural pneumonectomy or received chemotherapy. Multivariate analysis identified CD9 expression as an independent prognostic factor with a hazard ratio (HR) of 1.99 in the analysis of all mesotheliomas (P=0.0261) and an HR of 2.60 in the analysis of EMs (P=0.0376). CD9 expression is an independent favorable prognostic marker of malignant mesothelioma.

## Introduction

CD9, a 24- to 27-kDa cell surface glycoprotein, is a member of the tetraspanin superfamily. It is expressed in numerous normal tissues and plays a critical role in various types of cell processes, such as cell adhesion, motility and various signaling pathways involving integrins. In malignancies, its expression usually suppresses tumor progression and metastasis by inhibition of tumor proliferation and survival (1,2). Although converse functions have also been reported in certain tumors, downregulation of CD9 correlates well with tumor progression or metastasis in bladder, breast, lung and colon cancers (2). An *in vivo* study using administration of the CD9 antibody to mice bearing human gastric cancer xenografts showed inhibition of tumor progression via anti-proliferative, pro-apoptotic and anti-angiogenetic effects (3), suggesting its potential for the molecular-targeted therapy of human malignancies. Moreover, we previously identified CD9, along with side population, CD24 and CD26 cells, as a cancer stem cell marker of mesothelioma, thus demonstrating its potential for cancer stem cell-targeted therapy in the future (4).

Malignant mesothelioma is an aggressive cancer with few patients surviving beyond 2 years following diagnosis. The median survival of patients without any treatment barely exceeds 1 year. A large population-based study reported 6-month, 1-year and 5-year overall survival rates of 55, 33 and 5% in mesothelioma (5). In Japanese patients, the median survival of mesothelioma has been reported to be 9–10 months from the date of diagnosis (6,7).

The clinical predictors for poor survival in patients with mesothelioma are reported to include sarcomatoid histology, older age, advanced IMIG stage, patients without palliative surgery or chemotherapy. Other biological prognostic factors such as serum and tumor EGFR expression (8,9), pleural effusion VEGF level (10), angiopoietin-1 expression (11), ER-β expression (12), methylation profile (6,13) and miRNA signatures (14) have also been reported. In this study, we identified CD9 as an independent predictor of survival, and loss of expression showed biological aggressive behavior in mesothelioma cells.

## Materials and methods

### Cell line

Mesothelioma cell lines, MSTO-211H [derived from biphasic mesothelioma (BM)] and TUM1 (4) were maintained in RPMI-1640 medium (Gibco-BRL; Invitrogen Life Technologies, Grand Island, NY) supplemented with 10% fetal calf serum (FCS), 100 U/ml penicillin and 100 mg/ml streptomycin. The cells were maintained as monolayers in 10-cm diameter cell culture dish at 37°C in a humidified atmosphere of 5% CO_2_ in air.

### shRNA lentiviral transfection

CD9-targeted shRNA lentiviral plasmid (Mission; Sigma-Aldrich, target sequence: ccgggctg ttcggatttaacttcatctcgagatgaagttaaatccgaacagctttttg) and non-targeting control plasmid (pLKO.1-puro) were transfected with ViraPower™ Lentiviral packaging mix to cell lines using Lipofectamine 2000 (Invitrogen Life Technologies). The cells were transfected with the shRNA-expressing lentivirus, and stable cell lines were generated by selection with puromycin. Knockdown of CD9 was confirmed by FACS analysis with the FITC mouse anti-human CD9 antibody (BD Pharmingen).

### In vitro migration assay

Migration assay was performed using a 24-well Boyden chamber with a non-coated 8-mm pore size filter in the insert chamber (BD BioCoat). Cells (CD9 shRNA- and control shRNA-transfected MSTO-211H) (5×10^4^) were suspended in 0.5 ml RPMI-1640 media containing 0.1% FCS and seeded into the insert chamber. Cells were allowed to migrate for 48 h into the bottom chamber containing 1 ml of RPMI-1640 media containing 10% FCS in a humidified incubator at 37°C in 5% CO_2_. Migrated cells which had attached to the outside of the filter were visualized by staining with Diff-Quik (International Reagents Co.) and counted.

### Patients and tissue specimens

One hundred and twelve cases of malignant pleural mesothelioma were retrieved from the archival pathology files of the Department of Pathology, Graduate School of Biomedical Sciences, Hiroshima University. Small biopsy specimens were not included in this study. The histological diagnosis of mesothelioma was previously carried out by three independent pathologists (V.J.A., Y.T., K.I.) based on WHO criteria (15) and were confirmed in all instances by clinical, histological and immunohistochemical findings. Epithelioid mesothelioma (EM) was further subdivided into two subtypes, i.e., ‘differentiated’ type (EM-D) and ‘less-differentiated’ type (EM-LD) based on the morphology of ‘papillo-tubular structures’ as an indicator of differentiation (16). Thus, EM-D were EMs showing a papillo-tubular pattern, micropapillary pattern and/or microcystic pattern and EM-LD were EMs showing solid nest, trabecular pattern, signet-ring cell-like appearance and/or single-cell infiltration pattern. The histological classification was carried out prior to this study. The clinical data of the patients were retrieved from the hospital records. This study was carried out in accordance with the Ethical Guidelines for Epidemiological Research enacted by the Japanese Government as tissue specimens were collected and carried out strictly to protect personal identity after approval by the Institutional Review Board at Hiroshima University.

### Immunohistochemistry

Immunohistochemical stainings were performed on 3-μm paraffin sections using the monoclonal anti-CD9 antibody. Tissue sections were deparaffinized, hydrated and endogeneous peroxide was quenched using 0.3% hydrogen peroxidase for 30 min. Sections were incubated in a humidified chamber with mouse monoclonal anti-CD9 antibody (diluted 1:100, clone 72F6, NB110–41534; Novus Biologicals, Littleton, CO, USA) overnight at 4°C. The reaction was visualized using the Histofine Simple Stain kit (Nichirei Biosciences, Tokyo, Japan) with diaminobenzidine as a chromogen and nuclear counterstaining with Mayer’s hematoxylin. A similar immunohistochemical procedure was carried out with the omission of the primary antibody as a negative control. Endothelial cells in and around the tumor tissue were considered as the internal positive control for validation of the immunohistochemistry. The membranous staining of CD9 was scored as 0, no staining; 1^+^, 1–10%; 2^+^, 10–50%; and 3^+^, >50% of tumor cells immunostained in the tissue sections. Immunohistochemical scoring was carried out by two pathologists (V.J.A., Y.T.) independently without knowledge of the clinicopathologic or disease outcome variables. Multiple sections from different paraffin blocks were analyzed for CD9 expression to confirm negative CD9 expression.

### Statistical analysis

Fisher’s exact test and Pearson Chi-square test were used for association analyses of the clinicopathological parameters with CD9 expression. Univariate analysis and multivariate Cox proportional hazards regression analysis for overall survival were performed with the regressors: CD9 expression, age, gender, clinical IMIG staging, histological type, differentiation, therapeutic regimen, extrapleural pneumonectomy and chemotherapy status. The overall survival curves of patients with follow-up data were estimated using the Kaplan-Meier method. Multivariate Cox proportional hazard ratio (HR) was separately calculated for all mesotheliomas and EMs for patients with clinical data for all parameters including age, histology (in analyzing all cases)/differentiation (in analyzing EM alone), IMIG staging and therapeutic regimen. Statistical analysis of the migration assay was performed by the two-tailed t-test. All of the statistical analyses were carried out using JMP 9.0 software. P-value <0.05 was considered to indicate a statistically significant result.

## Results

### Effect of shRNA knockdown of CD9 on cell migration

**Figure 1:**
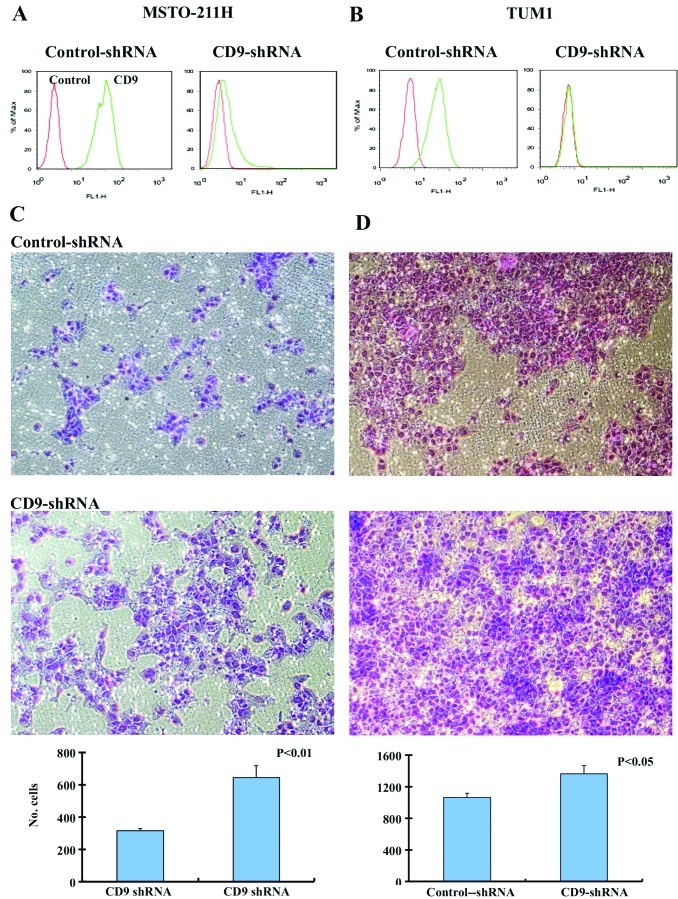
Flow cytometric analysis of CD9 expression in (A) MSTO-211H and (B) TUM1 cells transfected with control-shRNA and CD9-shRNA. Successful knockdown of CD9 was confirmed. Boyden chamber migration assay showed significantly increased cell migration of (C) MSTO-211H and (D) TUM1 cells transfected with CD9-shRNA (lower panels) in comparison to the control-shRNA (top panels).

Immunohistochemical analysis of CD9 expression in the patient mesothelioma samples indicated that CD9 expression was a favorable prognostic factor. Therefore, we investigated the effects of CD9 on the cell migration of mesothelioma cell lines using shRNA-mediated CD9 knockdown. Knockdown of CD9 in the MSTO-211H and TUM1 cell lines was confirmed by FACS analysis, and the migration of cells was analyzed using Boyden chamber assay (Fig. 1). CD9 shRNA-transfected and control shRNA-transfected MSTO-211H and TUM1 cells were allowed to migrate toward medium containing 10% FCS as a chemoattractant. CD9 shRNA-transfected MSTO-211H and TUM1 cells showed increased migration in comparison to the control shRNA-transfected cells (Fig. 1).

### Clinicopathological characteristics of the malignant mesothelioma patients

This study consisted of 103 male patients and 9 female patients (M:F ratio 11:1), with a mean age of 65.8 years with standard deviation of 9.4 (range 42–88 years). Twenty-five cases were IMIG stage I, 40 cases were stage II, 25 cases were stage III and 21 cases were stage IV. Histologically, 71 cases were EMs (63.4%) 21 cases were sarcomatoid mesothelioma (SM) (18.75%) and 20 cases were BM (17.85%). Thirty-three cases of EM were differentiated type (EM-D) and 38 cases were less differentiated type (EM-LD).

Clinically, 30 patients received best supportive care alone, 42 patients were treated with chemotherapy alone and 40 cases underwent extrapleural pneumonectomy with or without chemotherapy and/or radiotherapy. Other surgical procedures were not included in this study as their numbers were limited for the analysis. The chemotherapy regimen consisted of various combinations: pemetrexed plus cisplatin regimen (39 cases), pemetrexed plus carboplatin (5 case), pemetrexed-containing regimen (44 cases) and chemotherapy regimens without pemetrexed (30 cases). The mean follow-up period was 16.5 months (from 10 days to 79 months) with 94 patients succumbing to the disease and 18 alive with disease at the time of the study.

### CD9 expression in malignant mesothelioma

**Figure 2:**
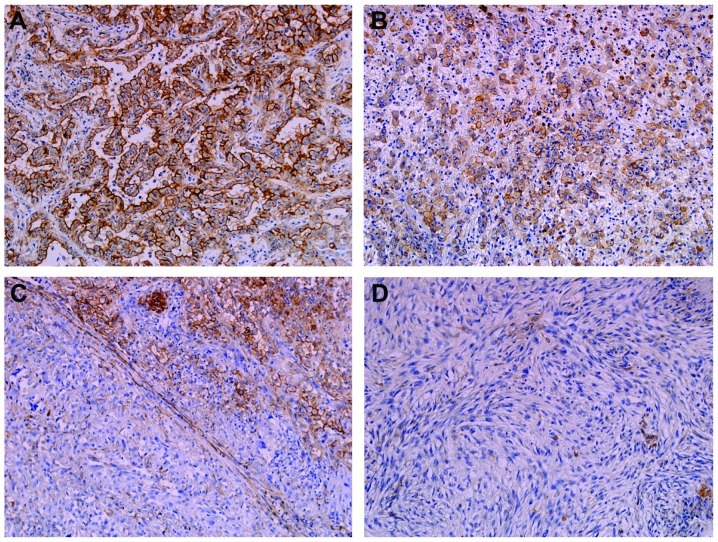
CD9 immunohistochemistry. (A) Epithelioid mesothelioma differentiated type showing diffuse CD9 expression. (B) Epithelioid mesothelioma less differentiated type showing decreased CD9 expression. (C) Biphasic mesothelioma showed CD9 expression in the epithelioid component while CD9 expression was not evident in the sarcomatoid component. (D) CD9 was not evident in the sarcomatoid component of sarcomatoid mesothelioma as well.

Positive immunoreactivity for CD9 was observed in the membrane and cytoplasm of the tumor cells in 76 of 112 malignant mesothelioma cases. Histologically, CD9 immunoreactivity was observed in 62 of 71 epithelioid mesotheliomas, 13 of 20 biphasic mesotheliomas and only 1 of 21 SMs. Among EMs, all cases of EM-D were CD9-positive and showed higher immunohistochemical score for CD9 expression compared to cases of EM-LD (Fig. 2) (Table I).

### Association between CD9 expression and clinicopathological parameters

To determine the statistical significance of CD9 expression in malignant mesothelioma, all cases were divided into two groups based on their CD9 expression: a CD9-positive (n=76, 67.9%) and a CD9-negative (n=36, 32.1%) group. The association between CD9 expression and various clinicopathological parameters is listed in Table II. Mesothelioma patients with a younger age (P=0.0083), epithelioid histology (P<0.0001), differentiated type EMs (P=0.0027), who did not receive best supportive care (P=0.0469), who underwent EPP and chemotherapy (P=0.0195) and who received chemotherapy with inclusion of pemetrexed (P=0.0434) showed statistically significantly a high frequency of positive CD9 expression.

### Association between CD9 expression and patient prognosis

**Figure 3:**
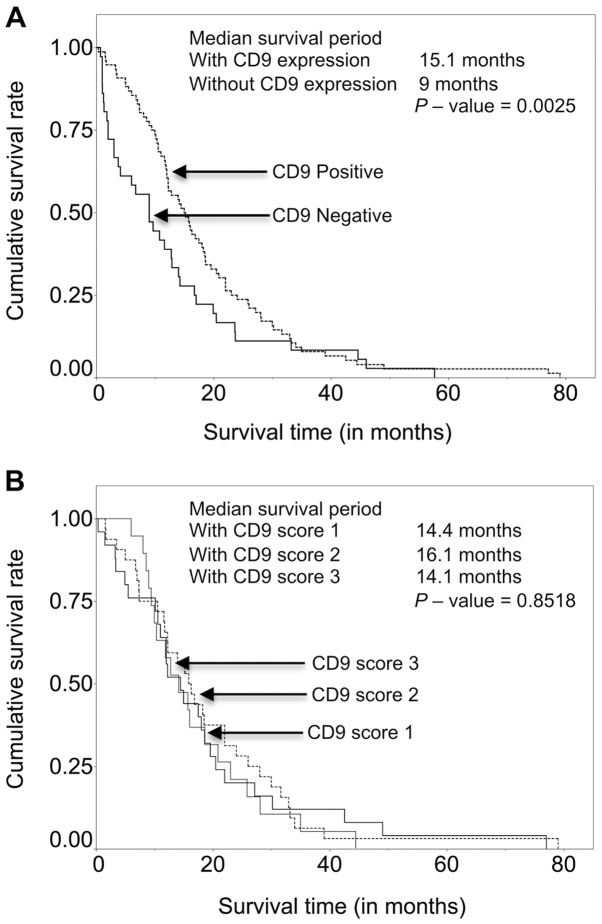
Kaplan-Meier curves showing overall survival of patients with mesothelioma in relation to CD9 expression status. (A) Patients with and without CD9 expression are represented by straight and dotted lines, respectively. (B) Patients with immunohistochemical score 1, 2 and 3 for CD9 expression showed no significance differences in overall survival.

The median survival period for the CD9-positive group was 15.1 months and that for the CD9-negative group was 9 months. The difference between the two groups was statistically significant (Wilcoxon; P=0.0025) (Fig. 3A). However, when CD9 expression was stratified according to immunohistochemical scores, no significant association was noted between CD9 expression score and patient survival (Fig. 3B). The CD9-positive group showed higher 1- and 2-year survival rates (63.2 and 25.0%) compared to the CD9-negative group (38.9 and 11.1%) (Table III).

**Figure 4:**
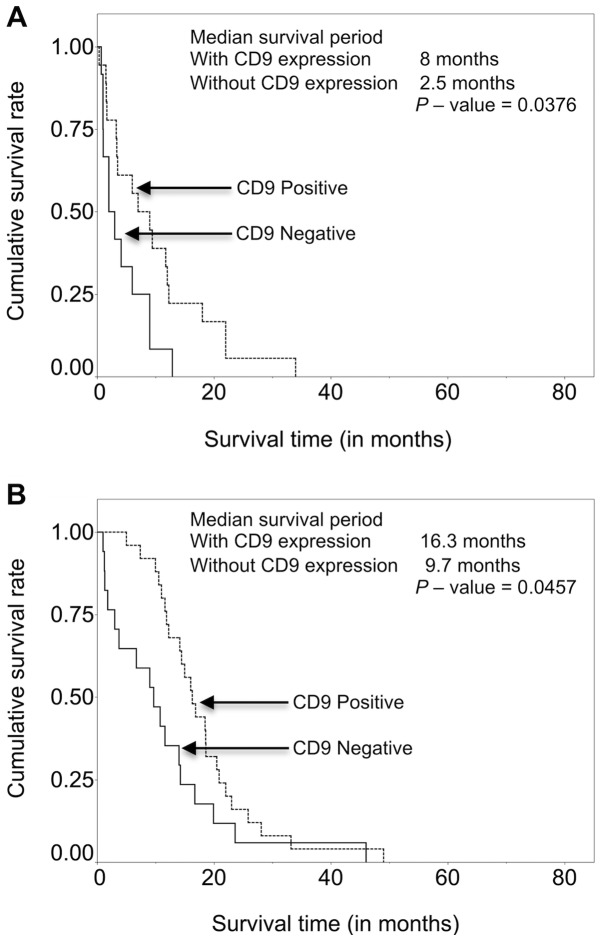
Kaplan-Meier survival curves showing overall survival of patients with mesothelioma with and without CD9 expression (A) receiving best supportive care and (B) receiving chemotherapy alone. Straight-line curve, without CD9 expression and dotted line curve, with CD9 expression.

Among the patients receiving best supportive care, patients with CD9 expression had higher survival (mean survival time 8 months) compared to those without CD9 expression (mean survival time, 2.5 months) (P=0.0376). A similar result was found among the patients treated with chemotherapy alone with a mean survival time of 16.2 months for patients with CD9 expression and 9.7 months for patients without CD9 expression (P=0.0037) (Fig. 4).

Other clinicopathological parameters that correlated significantly with overall survival according to univariate analysis (Table III) included age (P=0.0003), IMIG staging (P=0.0001), histology (P<0.0001), differentiation (P=0.0301), therapeutic regimen (P=<0.0001), extrapleural pneumonectomy (P<0.0001) and chemotherapy (P<0.0001). Chemotherapy with inclusion of pemetrexed showed a tendency for better survival, but did not achieve statistical significance (P=0.0715). Multivariate analysis using the Cox proportional hazards model of mesothelioma patients showed loss of CD9 expression as an independent predictor of overall survival in patients with malignant mesothelioma with an HR 1.99 (P=0.0261) in addition to age, IMIG staging, histology and therapeutic regimen (Table IV). As the CD9 expression in EMs showed a significant difference in the differentiation type, we also analyzed multivariate analysis using Cox proportional hazards model of 71 EMs. CD9 expression was again an independent predictor of overall survival with an HR of 2.60 (P=0.0376) along with other factors; age (P=0.0023) therapeutic regimens, chemotherapy (P=0.0113) and extrapleural pneumonectomy (P=0.0014), but not IMIG staging (P=0.1336) and differentiation (P=0.1337) (Table V).

## Discussion

Disruption of cell adhesion and alteration in cell motility play an important role in cancer cell invasion and metastasis. The tetraspanin superfamily proteins (TM4SF) mainly CD9, CD63, CD82, CD151 and CD81 have been implicated in cell migration, proliferation and tumor cell metastasis (17,18). CD9 is to date the best characterized member of the TM4SF proteins and is involved in cell growth, adhesion and motility. Moreover, CD9 has been recently reported as a prognostic factor in adenocarcinoma of the lung (19), colon (20), breast (21), pancreas (22), prostate (23) and SCC of the esophagus (24) and oral cavity (25). We found increased cell migration in CD9-knockdown mesothelioma cell lines. In this migration assay experiment using MSTO-211H cells, we found a decrease in CD9 expression after CD9-shRNA transfection which led to increased cell migration compared to control-shRNA-transfected cells.

These data suggest the importance of CD9 in determining the aggressive behavior of malignant mesothelioma. Recently, Nakamoto *et al*(3) investigated the antitumor effect of the anti-CD9 monoclonal antibody (ALB6) in human gastric cancer cell xenografts. They found a profound effect on tumor progression by anti-proliferative, pro-apoptotic and anti-angiogenetic effects. Moreover, we previously identified CD9 along with side population CD24 and CD26 cells to be markers of cancer stem cells in mesothelioma. We also demonstrated that CD9-positive cell lines had a clear tendency to generate larger tumors in mice (4). Thus, CD9 may be a potential candidate as a molecular target in the treatment of mesothelioma.

Loss of CD9 expression correlates with poor prognosis in bladder carcinoma (26) and esophageal squamous cell carcinoma (24), small and non-small cell lung cancers (27,28) and prostatic carcinoma (23). This study is the first to analyze CD9 expression in human mesothelioma tissue and to correlate its expression with survival with other clinicopathological parameters. CD9 expression was noted more frequently in younger patients, IMIG stage I–II and epithelioid histology compared to older patients, IMIG stage III–IV and sarcomatoid histology.

The present study found that the loss of CD9 expression in mesothelioma is related to a shorter overall survival (medial survival 9 months, 1-year survival 38.9% and 2-year survival 11.1%) compared to patients with CD9 expression (median survival 15.1 months, 1-year survival, 63.2% and 2-year survival 25%). When CD9 expression in mesothelioma was stratified according to score (1–3) did not show a statistically significant association with overall survival rates (Fig. 3B), suggesting that the complete loss of CD9 expression has more significance than the extent of CD9 expression. Age, IMIG staging, histology, differentiation of epithelioid mesothelioma, therapeutic regimen, status of extrapleural pneumonectomy and status of chemotherapy all had a statistically significant association with overall survival. Patients with CD9 expression had higher survival compared to those without CD9 expression in patients receiving best supportive care or patients treated with chemotherapy. This suggests the importance of CD9 expression as an indicator for patients receiving chemotherapy in mesothelioma patients.

The multivariate analysis showed CD9 expression is an independent predictor of survival of mesothelioma patients with an HR of 1.99 (P=0.0261) as well as older age (HR, 2.10), IMIG stage III/IV (HR, 2.04), sarcomatoid histology (HR, 6.65), patients treated with chemotherapy alone (HR, 0.37) and patients treated with extrapleural pneumonectomy (HR, 0.26) (Table IV). As CD9 expression was observed in only one case of sarcomatoid mesothelioma and sarcomatoid histology was itself a strong independent predictor of survival of mesothelioma patients, multivariate analysis excluding sarcomatoid mesothelioma is necessary to evaluate the importance of CD9 expression as an independent predictor of mesothelioma survival. Hence, we performed multivariate analysis of patients with epithelioid mesothelioma alone and we again found that CD9 expression was a predictor of survival of mesothelioma patients with an HR of 2.60 (P=0.0376) (Table V), suggesting the independent prognostic value of CD9 expression in mesothelioma.

In the present study, sarcomatoid mesothelioma did not show CD9 expression. It may thus be hypothesized that the loss of CD9 expression in epithelioid mesothelioma leads to loss of epithelioid differentiation and ultimately transition into sarcomatoid mesothelioma. This is supported, in part, by the fact that histologically less-differentiated epithelioid mesotheliomas showed lower CD9 expression. In contrast, loss of differentiation or epithelial-mesenchymal transition by other molecular pathways leading to loss of CD9 expression may also be postulated. The biological significance of loss of CD9 expression in sarcomatoid mesothelioma requires further investigation.

In conclusion, CD9 expression is a favorable prognostic marker in patients with mesothelioma. Our *in vitro* study demonstrated increased cell migration after CD9 knockdown, suggesting loss of CD9 as a predictor of more aggressive behavior. CD9 expression may be also an indicator of epithelial-mesenchymal transition from epithelioid mesothelioma to sarcomatoid mesothelioma.

## Figures and Tables

**Table I:** Immunohistochemical scoring of CD9 expression in malignant mesothelioma.

		CD9 immunohistochemical scoring			
		
Histology	Total cases	0	1	2	3
Epithelioid mesothelioma	71	9	21	25	16
Differentiated type	33	0	6	15	12
Less-differentiated type	38	9	15	10	4
Sarcomatoid mesothelioma	21	20	0	1	0
Biphasic mesothelioma	20	7	4	6	3

**Table II:** Clinicopathological characteristics of patients with mesothelioma and its correlation with CD9 expression.

	CD9 expression			
		
Clinicopathological parameters	Total cases	Positive	Negative	P-value^b^
Age (years)				
<60	35	30	5	0.0083
≥60	77	46	31	
Gender				
Male	103	68	35	0.2672
Female	9	8	1	
IMIG staging				
Stage I/II	65	48	17	0.1511
Stage III/IV	47	28	19	
Histology				
Epithelioid	71	62	9	<0.0001^c^
Sarcomatoid	21	1	20	
Biphasic	20	13	7	
Differentiation in epithelioid mesothelioma				
Differentiated	33	33	0	0.0027
Less-differentiated	38	29	9	
Therapeutic regimen				
BSC alone	30	18	12	0.0469^c^
Chemotherapy alone	42	25	17	
Extrapleural pneumonectomy^a^	40	33	7	
Extrapleural pneumonectomy (EPP)				
With EPP	40	33	7	0.0195
No EPP	72	43	29	
Chemotherapy				
With/without EPP and/or RT	74	50	24	1.0000
No chemotherapy	38	26	12	
Chemotherapy				
With pemetrexed	44	34	10	0.0434
Without pemetrexed	30	16	14	

IMIG, International Mesothelioma Interest Group; BSC, best supportive care; RT, radiotherapy.

a This group consisted of patients receiving EPP alone or EPP with chemotherapy and/or radiotherapy.

b Two-tailed Fisher’s exact test.

c Pearson’s Chi-square test.

**Table III:** Univariate analysis of overall survival in patients with malignant mesothelioma.

	Survival in months				
				
Clinicopathological parameters	Median	Mean	1-year survival	2-year survival	P-value
Age (years)					
<60	22.0	25.4	77.1%	42.9%	0.0003
≥60	11.6	12.6	45.5%	10.4%	
Gender					
Male	12.2	15.3	53.4%	18.5%	0.0789
Female	18.0	30.7	77.8%	44.4%	
IMIG staging					
Stage I/II	16.3	20.4	63.5%	30.2%	0.0001
Stage III/IV	9.0	9.9	42.4%	3.1%	
Histology					
Epithelioid	15.7	19.5	63.4%	29.6%	<0.0001
Sarcomatoid	3.8	6.1	14.3%	0%	
Biphasic	13.9	17.0	70.0%	10.0%	
Differentiation in epithelioid mesothelioma					
Differentiated	18.6	22.5	72.7%	36.4%	0.0301
Less differentiated	14.0	17.0	55.3%	23.7%	
Therapeutic regimen					
BSC	5.1	7.7	23.3%	3.3%	<0.0001
Chemotherapy alone	14.2	15.3	57.1%	11.9%	
Extrapleural pneumonectomy	18.9	24.5	77.5%	42.5%	
Extrapleural pneumonectomy					
With EPP	18.9	24.5	77.5%	42.5%	<0.0001
Without EPP	11.0	12.1	43.1%	8.3%	
Chemotherapy					
With/without EPP and/or RT	16.5	19.7	70.3%	27.0%	<0.0001
No chemotherapy	6.4	11.0	26.3%	7.9%	
Chemotherapy					
With pemetrexed	18.4	21.2	81.8%	29.6%	0.0715
Without pemetrexed	13.4	17.4	53.3%	23.3%	
CD9 expression					
Positive	15.1	18.4	63.2%	25.0%	0.0025
Negative	9	12.6	38.9%	11.1%	

IMIG, International Mesothelioma Interest Group; BSC, best supportive care; EPP, extrapleural pneumonectomy; RT, radiotherapy.

**Table IV:** Multivariate analysis of overall survival in malignant mesothelioma (Cox proportional hazards model).

		95% confidence interval		
			
Prognostic factors	Hazard ratio	Lower	Upper	P-value
CD9 expression	1.99	1.08	3.82	0.0261
Age, 60 years or more	2.10	1.24	3.66	0.0053
IMIG stage III/IV	2.04	1.23	3.37	0.0059
Histology against epithelioid mesothelioma				
Biphasic mesothelioma	2.13	1.15	3.87	0.0171
Sarcomatoid mesothelioma	6.65	2.91	15.22	<0.0001
Therapeutic regimen against BSC				
Chemotherapy alone	0.37	0.21	0.67	0.0011
Extrapleural pneumonectomy	0.26	0.14	0.50	<0.0001

BSC, best supportive care; IMIG, International Mesothelioma Interest Group.

**Table V:** Multivariate analysis of overall survival in epithelioid mesothelioma (Cox proportional hazards model).

		95% confidence interval		
			
Prognostic factors	Hazard ratio	Lower	Upper	P-value
CD9 expression	2.60	1.05	7.37	0.0376
Age, 60 years or more	2.62	1.40	5.12	0.0023
IMIG stage III/IV	1.64	0.85	3.11	0.1336
Less-differentiated type	1.54	0.87	2.71	0.1337
Therapeutic regimen against BSC				
Chemotherapy alone	0.37	0.18	0.79	0.0113
Extrapleural pneumonectomy	0.27	0.12	0.59	0.0014

BSC, best supportive care; IMIG, International Mesothelioma Interest Group.
